# Refugees and COVID-19: achieving a comprehensive public health response

**DOI:** 10.2471/BLT.20.271080

**Published:** 2020-08-01

**Authors:** Qais Alemi, Carl Stempel, Hafifa Siddiq, Eunice Kim

**Affiliations:** a School of Behavioral Health, Department of Social Work & Social Ecology, Loma Linda University, 1898 Business Center Drive, San Bernardino, CA 92408, United States of America (USA).; b Department of Sociology and Social Services, California State University, Hayward, USA.; c The National Clinician Scholars Program, University of California, Los Angeles, USA.

Millions of refugees worldwide are exposed to violence, family separation, culture loss and exile. The coronavirus disease 2019 (COVID-19) exposes these populations to a new threat, one that could prove to be more devastating than the events forcing them to flee their homelands.

Refugees are vulnerable to COVID-19,^1^ as they live in conditions that disproportionately increase their risk of contagion. For example, in densely populated refugee camps, social distancing is challenging and if basic sanitation is lacking, proper hand hygiene is close to impossible.^2^ Projections in Cox’s Bazar, Bangladesh, which hosts over 600 000 Rohingya refugees, suggest that a COVID-19 outbreak could exhaust medical resources and overwhelm camp hospitals within 58 days, which would lead to a rise in deaths from other infectious diseases, such as malaria.^2^ Although limited evidence exists on whether infectious diseases increase the risk of COVID-19, the World Health Organization anticipates that people who have both COVID-19 and other infectious diseases, such as tuberculosis, may have poorer treatment outcomes, especially if tuberculosis treatment is interrupted.^3^ This prediction is alarming, considering that tuberculosis and malaria are highly prevalent in refugee populations, as are noncommunicable diseases, such as type 2 diabetes,^4^^,^^5^ known to increase susceptibility to severe COVID-19. This situation is compounded by language barriers that refugees face in host communities and their limited access to health care for obtaining health information, testing and treatment, which some may even avoid out of fears of being deported.^4^

The refugees’ fear of being isolated in quarantines and separated from their families, or even killed to slow the pandemic, can explain why aid workers in Rohingya camps report minimal testing among residents with COVID-19 symptoms.^6^ COVID-19-related stigmatization adds to this challenge. Fears of refugees in the general public may be compounded by COVID-19 fears, increasing discrimination against these groups. The social stigma associated with COVID-19 may encourage illness concealment, delay early detection and treatment, increase distrust in health authorities, lower the likelihood of compliance and prolong recovery.^7^

While empirical studies are needed to understand the extent and nature of stigma in refugees with COVID-19, news media reports from refugee-sending countries, such as Iraq, indicate that stigma is a major barrier to prevention and treatment.^8^

Moreover, the COVID-19 pandemic has strained the finances of governments, nongovernmental organizations and humanitarian agencies that serve refugees. The economic crisis caused by efforts to contain the pandemic is worsening the refugees’ already precarious situation in informal labour markets. Without access to government support for unemployed citizens, many refugees rely on insufficient cash assistance from humanitarian agencies and many will not have jobs waiting for them when business reopens. The United Nations High Commissioner for Refugees reports that a recent survey in Jordan showed that only 35% of Syrian refugees said they had a secure job to return to after the lifting of COVID-19 restrictions.^9^ This economic hardship might interact with past trauma exposure to prolong and exacerbate mental health conditions in refugee populations.^10^ Mental health conditions may also become a barrier to accurate personal risk assessment and use or maintenance of COVID-19-related precautionary practices.^7^

The COVID-19 pandemic has exposed systems of inequality but was met with delayed responses by public health authorities to address the needs of the most vulnerable. Humanitarian agencies serving refugees emphasize the importance of global support for the receiving countries so they can continue their efforts of solidarity, medical care and economic support. Hence, we strongly endorse *The Lancet*’s guiding principle of public health networks leaving no one behind during the COVID-19 pandemic,^11^ recommending epidemiologic risk assessments and the timely deployment of outbreak response teams within refugee camps, promoting health education in a culturally sensitive manner and ensuring health care access without refoulment for refugees.

As resources are constrained, agencies and professionals serving refugees should consider giving priority to screening vulnerable subgroups (that is, those with chronic health conditions) to manage comorbidities more effectively, encourage patients to reduce high-risk behaviour, provide treatment and reduce transmission rates.^12^ Moreover, professionals serving refugees in camps and in host communities should expect that stigma will influence preventive measures and treatment-seeking among refugees, and should therefore consider encouraging care providers and local leaders to dispel fears, misconceptions and the stigma associated with COVID-19. Lastly, overlooking mental health conditions, exacerbated by the socioeconomic hardship caused by this pandemic, will complicate refugees’ integration and increase the uncertainty they endure. Governments, public health professionals and organizations should act now to prevent the spread of COVID-19 in refugees whose vulnerabilities place them at great risk of mortality.
